# Remote Longitudinal Tracking of Sleep, Depression, and Cognition Across Early Adolescence: Protocol for a Longitudinal Observational Study

**DOI:** 10.2196/86345

**Published:** 2026-10-06

**Authors:** Katharine C Simon, Lia Galut, Spencer Polk, Shun Iwata, Weining Shen, Mahyar Abbasian, Amir Rahmani, Uma Rao, Jessica L Borelli, Sara C Mednick

**Affiliations:** 1 Department of Pediatrics, University of California, Irvine, School of Medicine, 5141 California Ave, Irvine, CA, 92697, United States, 1 8054534462; 2 Pulmonology Department, Rady Children’s Health, Children’s Hospital of Orange County, Orange, CA, United States; 3 Department of Statistics, University of California, Irvine, Irvine, CA, United States; 4 Department of Computer Science, University of California, Irvine, Irvine, CA, United States; 5 School of Nursing, University of California, Irvine, Irvine, CA, United States; 6 Deparment of Psychiatry and Human Behavior, University of California, Irvine, School of Medicine, Irvine, CA, United States; 7 Department of Psychology, University of California, Irvine, Irvine, CA, United States; 8 Department of Cognitive Science, University of California, Irvine, Irvine, CA, United States

**Keywords:** depression, sleep, cognition, development, remote research, mobile health, wearable technology

## Abstract

**Background:**

Depression in adolescents is a growing public health concern, with 3.5 million youth in the United States experiencing a depressive episode each year. When symptoms emerge in early adolescence, they can have lasting psychological, physical, and cognitive consequences, highlighting the urgent need for proactive intervention. Insufficient sleep—characterized by short duration, irregular timing, and poor quality—is bidirectionally linked to depression and associated with impaired cognitive performance.

**Objective:**

This protocol provides the map for a study tracking sleep patterns and neurophysiology, as well as a range of depressive symptoms, and their dynamic interaction in shaping longitudinal cognitive trajectories.

**Methods:**

Fifty-six youth aged 9 to 13 (mean 11.01, SD 1.39) years were enrolled in a longitudinal measurement burst design comprising 4 assessment waves at baseline, 4, 8, and 12 months. We recruited a sample of youth with a range of depressive symptom severity. Each wave included 7 consecutive days of monitoring during which participants used a personalized mobile health (mHealth) platform, HowRU app, developed by the study team, to report daily sleep patterns, thrice-daily ecological momentary assessments (EMAs), and completed a series of cognitive tasks. Youth and parents also completed validated psychosocial questionnaires to assess mood, behavioral concerns, mental health, and sleep. Sleep and physical activity were monitored remotely using a Garmin VivoSmart 5 watch and Interaxon Muse S headbands. Sleep-dependent cognitive performance was assessed with 2 declarative memory tasks (Word-Pair Associates task [WPA] and Minecraft Memory and Navigation [MMN] task), a procedural memory task (Motor Sequence task), and 2 executive functioning tasks (Operation Span [OSpan] task and Rule Switch task [RST]). For the memory tasks, participants completed training and immediate testing in the evening, followed by a retention test the following morning. The OSpan task and Rule Switch were administered twice daily (morning and evening). For all tasks, stimuli were randomized and counterbalanced across participants and testing sessions.

**Results:**

Funding was obtained from the Eunice Kennedy Shriver National Institute of Child Health and Human Development (grant K08 HD107161), with a budget period of August 1, 2022, to July 31, 2027. Participant enrollment occurred May 2023 through October 2024. Data collection began in July 2023 and was completed in October 2025. Study participant retention and adherence rates for sleep diaries, cognitive tasks, and questionnaires were high. Primary outcomes of the study are anticipated to be published in fall 2026.

**Conclusions:**

This protocol provides a robust and reproducible framework to longitudinally track youth with a range of depressive symptoms in the real world using mHealth platforms and wearable technology. Potential findings can support the identification of key developmental periods of vulnerability and resilience in youth, inform future sleep-based interventions to promote mental health, and optimize cognitive outcomes across early adolescence.

## Introduction

Adolescent depression is a growing public health concern in the United States, with 5 million teens, equivalent to approximately 20% of adolescents, experiencing at least 1 major depressive episode [1-3]. Adolescence brings rapid biopsychosocial changes, including shifts in sleep patterns, circadian rhythm delay, brain development, and social independence, rendering this time period a window of susceptibility for the onset of depression [4,5]. Early depressive episodes confer increased risk for future depressive episodes, psychiatric and medical comorbidities, and suicide [1,2,6-8]. Depression in adolescence is also linked to cognitive impairments, particularly in executive function, inhibitory control, attention, and hippocampal-dependent memory [9]. These deficits can persist despite symptom remission and are associated with poorer treatment outcomes and elevated relapse risk [10-14]. The hippocampus, crucial for binding the episodic and spatial elements of new learning, undergoes structural and functional maturation during the transition to adolescence [15-18]. Depression can disrupt hippocampal development [19,20], possibly contributing to the memory impairment associated with depressive disorders.

Although the brain mechanisms underlying depression-related hippocampal-dependent memory deficits remain unclear, poor sleep may be a key risk factor. Sleep plays a critical role in the transformation of recent experiences into stable memories [21]. Specific features of nonrapid eye movement (NREM) sleep, such as slow wave sleep (0.1‐0.4 Hz) and spindles (12‐15 Hz), are particularly important for hippocampal-dependent memory consolidation [22,23]. In adults, sleep disturbances and deprivation lead to hippocampal dysfunction and poor memory [24,25]. In adolescents, short-term sleep restriction studies show mixed results: some demonstrate resilience to sleep loss, while others show deficits across cognitive domains [26-29]. Interestingly, despite these mixed findings regarding the behavioral manifestations of sleep loss, the physical manifestations of chronic insufficient sleep have consistently been shown to include altered brain structure, specifically reduced hippocampal volume, disrupted prefrontal cortex circuitry, and alterations to white matter development [24,30-35]. The lack of longitudinal studies examining sleep and memory during the adolescent transition contributes to this knowledge gap.

The relationship between depression and sleep is bidirectional, with sleep disturbances often predicting the severity of depressive symptoms over time [36-38]. Adolescent biological and neural maturation coincides with significant shifts in sleep patterns, including delayed bedtimes, reduced sleep duration, increased sleep onset latency, and diminished homeostatic sleep pressure [4,5,39]. Depression exacerbates these sleep disruptions in adolescents, increasing sleep onset latency, the frequency of arousals, and disturbances [36,40], and altering NREM sleep brain rhythms, including lower amplitude slow-wave activity [41-44]. Given the critical role of NREM sleep in hippocampal-dependent memory consolidation, depressed youth may be particularly vulnerable to cognitive deficits in this domain over time. This underscores the need for real-world longitudinal monitoring to better understand how the interplay between sleep disturbances and depression affects hippocampal memory development across adolescence.

Importantly, sleep is a modifiable risk factor, and validated interventions can increase sleep duration, reduce variability in sleep patterns, and, critically, lead to parallel improvements in depressive symptoms [36-38]. Few biomarkers have the potential to longitudinally track depression as effectively as sleep in real-world environments [45,46], and yet, this developmental window remains understudied largely due to challenges in conducting repeated, naturalistic assessments in early adolescence [47]. Using personalized mobile health (mHealth) platforms increases the accessibility and feasibility of participating in longitudinal research. Although more common in adults, access to technology is high in youth and has strong potential to reshape the ability to engage youth in longitudinal research. Further, the use of wearables to track sleep patterns, physiology, and physical activity in naturalistic settings offers a robust platform to investigate mechanistic questions of sleep and depression on cognition in large-scale longitudinal studies [48,49]. Understanding how sleep, depressive symptoms, and cognition interact over time is essential for developing precision medicine targets and integrating them into real-world, scalable interventions.

The current protocol provides a framework for tracking the daily and longitudinal dynamical associations between insufficient sleep and depressive symptoms on hippocampal-dependent memory trajectories in young adolescents 9 to 13 years. Longitudinal, measurement burst study designs are rare in sleep and cognition research, as most studies rely on short-term experiments lasting only a few days or weeks [47]. Further, longitudinal studies using measurement burst designs are notably absent during the transition from childhood to adolescence, a developmental window marked by heightened vulnerability for depressive symptom onset and characterized by pubertal development, circadian shifting, and hippocampal maturation. Leveraging mHealth platforms with integrated wearables, our study was designed to shed light on mechanistic pathways linking sleep and depressive symptoms and cognitive outcomes. By capturing the day-to-day intraindividual variability alongside longer-term change, this longitudinal approach has the potential to disentangle the relative contributions of sleep and depressive symptoms during these critical early stages of adolescent development.

## Methods

### Study Design and Setting

A measurement burst design was used to track day-to-day and longitudinal changes in sleep, depressive symptoms, and cognition in participants. Participants completed data collection at the following measurement bursts: baseline, 4 months, 8 months, and 12 months. Each burst was comprised of 7 consecutive days during which sleep, mood, and cognitive tasks were monitored, referred to as a study week (Figure 1 for study timeline). Youth and parents completed psychosocial questionnaires on mood, behavior, and sleep. Sleep and physical activity were monitored remotely using a Garmin VivoSmart5 watch and MuseS headband worn by the participant.

**Figure 1.**
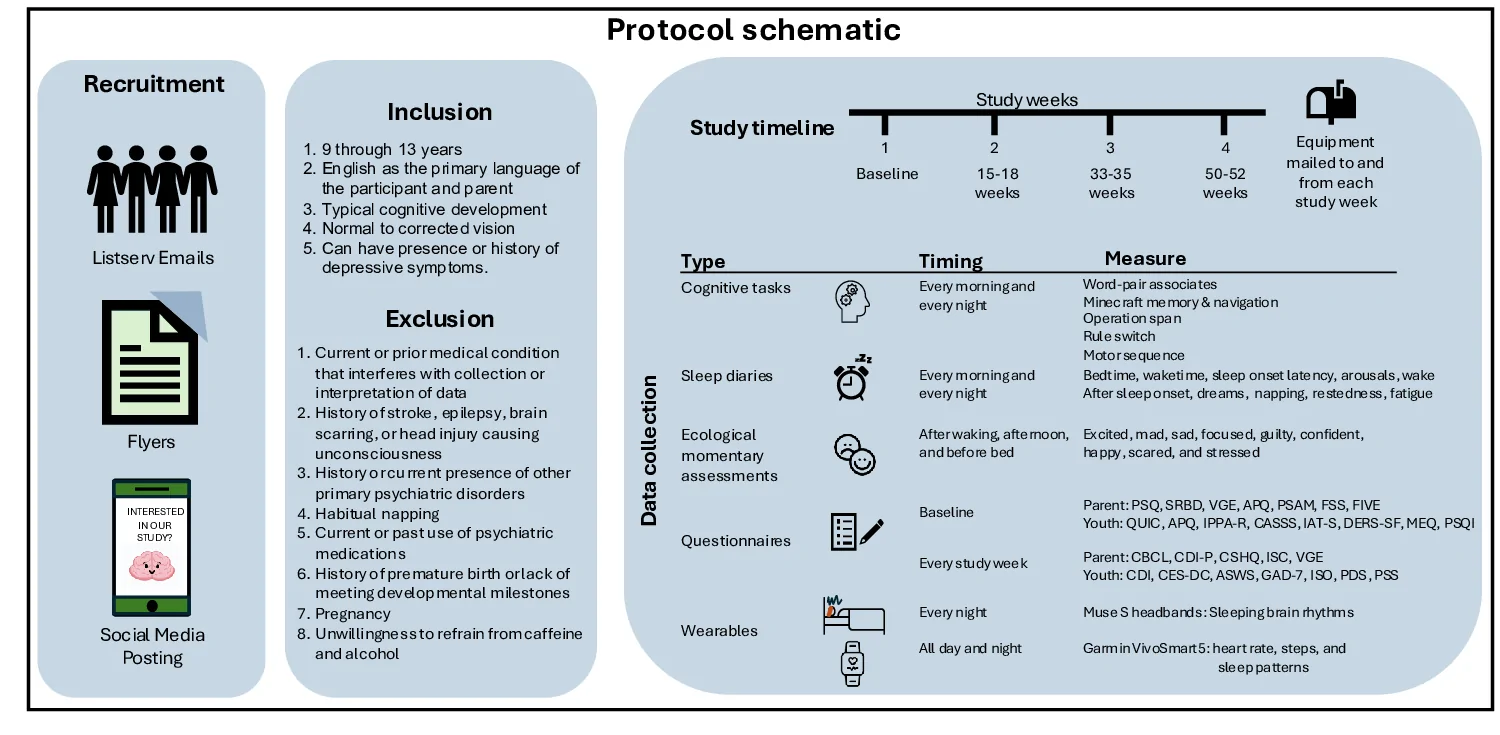
Study protocol schematic. Participants completed study week assessments approximately every 4 months. Each study week, participants completed daily cognitive tasks, ecological momentary assessments (EMA), and sleep diaries. Participants also used wearables to track sleep and wake patterns, physical activity, heart rate, and sleep neurophysiology. See baseline testing and measurement burst questionnaires for psychosocial questionnaires. APQ: Alabama Parenting Questionnaire; ASWS: Adolescent Sleep-Awake Scale; CASSS: Child and Adolescent Social Support Scale; CBCL: Child Behavior Checklist; CDI: Children’s Depression Inventory; CDI-P: Children’s Depression Inventory Parent; CES-DC: Center for Epidemiological Studies Depression Scale for Children; CSHQ: Children’s Sleep Habit Questionnaire; DERS-SF: Difficulties in Emotional Regulation; FIVE: fear of illness and virus evaluation; FSS: Family Stressors Scale; GAD-7: General Anxiety Disorder-7; IAT-S: Internet Addiction Test- Short Form; IPPA-R: Inventory of Parent and Peer Attachments-Revised; ISC: inclusion of self and child; ISO: inclusion of self and others; MEQ: Morningness-Eveningness Questionnaire; PDS: Pubertal Development Scale; PSAM: Parenting Self Agency Measure; PSQ: Pediatric Sleep Questionnaire; PSQI: Pittsburgh Sleep Quality Index; PSS: Perceived Stress Scale; QUIC: Questionnaire of Unpredictability in Childhood; SRBD: sleep-related breathing disorder; VGE: video game experience.

### Participants

Participants from southern California and the western United States were eligible for the study if they met the following inclusion criteria: (1) aged 9 to 13 years, (2) English as the primary language of the participant and parent, (3) typical cognitive development and normal to corrected vision, and (4) could have presence or history of depressive symptoms. We recruited participants whose parents reported a range of depressive symptom severity, from minimal to moderate, on the screener. Participants with anxiety as the sole psychiatric diagnosis were excluded. Additional exclusion criteria included (1) current or prior medical condition that could interfere with the collection or interpretation of data; (2) history of stroke, epilepsy, brain scarring, or head injury causing unconsciousness; (3) presence or history of anxiety symptoms were allowed, but no other history or current presence of psychiatric disorders (eg, eating disorder, attention deficit hyperactivity disorder, and sleep disorder); (4) no evidence of habitual napping; (5) no current or past use of psychiatric medication; (6) no history of premature birth or lack of meeting developmental milestones; (7) pregnancy; and (8) unwillingness to refrain from caffeine and alcohol during quarterly study evaluations. These inclusion and exclusion criteria were used to maintain a relatively homogenous sample in this age range.

Participants were recruited using multiple methods, including web-based social media postings, listserv emails, and website posts, as well as flyers posted at hospitals, psychology and psychiatry offices, schools, and community centers. While we primarily recruited from Southern California and the Western States, participants were not excluded based on geographic location if they were within the United States.

### Ethical Considerations

This study was approved by the Institutional Review Board (IRB) at the University of California, Irvine (IRB# 20216879). In accordance with STROBE (Strengthening the Reporting of Observational Studies in Epidemiology), a checklist is included in Checklist 1. Youth participants and their parents received monetary compensation for participation. Youth participants were compensated a total of US $400 for completing all assessments at all visits, which included bonuses for timely completion of sleep diaries, ecological momentary assessments (EMAs), and cognitive testing. For each assessment wave, participants were guaranteed to receive US $30 for attendance, with additional task completion garnering additional daily compensation. In total, participants could receive up to US $75 for participating in the entire measurement burst. To enhance completion of daily testing, if participants completed more than 5 out of 7 days of sleep diaries, EMAs, and cognitive tasks, they could earn an additional US $15 per day. Bonus compensation was based on completing at least the morning sleep diary and 1 cognitive task on days 4 through 7. At study completion, participants received a US $100 bonus for completing the entire study. Given that daily participation took approximately 1 hour per day, this incentive structure was not considered undue influence on youth participants and appropriately accounted for time required to participate.

Parents were compensated a total of US $120 throughout the study, as they were responsible for completing parent-specific study questionnaires regarding youth’s sleep, psychological and social relationships, monitoring wearable device use and safety, and supporting participants in completing daily tasks. Parents received US $10 automatically for each study week for assisting the youth in using the wearables, plus an additional US $20 bonus for completing all parent-specific questionnaires. Compensation for participants and parents was provided at the end of each study week. For participants who withdrew, compensation was provided for completed study activities.

### Study Timeline

Participants enrolled in the study were remotely monitored over 1 year, with quarterly measurement bursts (Figure 1 outlines the study timeline). During each burst, participants were monitored for 7 consecutive days using our personalized mHealth app, HowRU, and wearable technology. On the HowRU app, participants completed daily sleep diaries, cognitive tasks, responded to thrice-daily EMAs, and completed psychosocial questionnaires. Across each assessment burst, participants wore a Garmin VivoSmart5 watch all day and night to objectively measure their sleep and wake and physical activity patterns. Participants also wore a MuseS electroencephalogram (EEG) headband to monitor sleep oscillations at night (InterAxon Inc). The Minecraft Memory and Navigation (MMN) task was administered on the first and last day of the measurement burst study week. Measurement burst start days were counterbalanced across weekdays and weekends. Bursts began randomly across school years and summer holidays. Scheduling was coordinated to accommodate holidays and vacations.

As this was a remote study, participants completed all experimental activities at home. To accomplish this remote design, home addresses were collected from parents at consent to mail equipment, and return address labels were provided. For each measurement burst, all materials for participating were provided, including a Samsung TabA9 preloaded with the HowRU, Garmin, LabFront, and Interaxon Muse apps required for participating, the MuseS headband, the Garmin VivoSmart5 watch, and paper-based questionnaires that were not licensed for mHealth platform administration (see Remote mHealth Platform section). Materials were securely mailed to parents and returned by mail directly to the research lab. For all research activities, daily tasks were broken down into manageable time blocks and took approximately 1 hour in total to complete. Daily tasks were distributed across the day as follows: in the morning, participants completed the sleep diary and EMA in an integrated diary (~5 minutes), followed by the cognitive tasks (~20 minutes). In the afternoon, participants completed the EMA (~3 minutes) jittered within a 2-hour time window. In the evening, participants completed cognitive tasks (~30 minutes), followed by the sleep diary and EMA (~5 minutes).

### Remote mHealth Platform

We developed the HowRU app, a mHealth platform built on the University of California, Irvine Zotcare platform [50], which we used in the current study. Our app included all task components, including sleep diaries, cognitive tasks, EMAs, and psychosocial questionnaires. App reminders were built into the platform to support task completion, sleep diary entry, and questionnaire completion. App reminders were personalized and sent to participants based on their personal sleep and wake patterns. Notification reminders were sent 1 hour before the typical bedtime and 30 minutes after the typical wake time. App reminders repeated every 30 minutes for up to 2 hours until tasks, diaries, or mood checks were completed. This meant that, on the home screen, a message would appear reminding the participant to complete the specific task. While reminders could be ignored, there were no options to opt out or snooze them. All data collected in the app were deidentified, encrypted, and stored on lab-based, password-protected servers. The app ran on a custom platform with no third-party access. Research staff monitored completion of the HowRU task, diary, and questionnaire daily and sent reminder emails to parents and participants to complete tasks as needed.

HowRU is hosted on the Zotcare platform, which serves as the central, deidentified data orchestrator for this research (Figure 2) [50]. Its architecture is specifically designed to decouple personal identity from cognitive and behavioral metrics, ensuring high-integrity research while maintaining strict compliance with US federal and state regulations for minors. This is a custom mobile app with encryption standards including in-transit Transport Layer Security (TLS) 1.2/1.3 Advanced Encryption Standard (AES)-256 and at-rest AES-256 bit encryption, and operated within a secure, virtual private cloud (VPC)-isolated cloud environment for data storage. On Zotcare, platform account ownership is researcher-based, with pregenerated accounts, and participants were assigned to the platform using unique subject-identifying codes. We use RESTful API with token-based authentication and exchange only deidentified JSON payloads. Zotcare uses a zero-protected health information policy (zero-PHI), meaning that no names, birthdays, or contact information were stored. The unique study identification number linking the participant number and identity was stored securely on an external, institutional database that was password-protected and inaccessible to the Zotcare system. Access was restricted via role-based access, such that the research team had a dashboard that could download granular game logs mapped only to specific participant identification numbers. System administrators had blind access for infrastructure maintenance but could not reidentify participants. Lastly, data were retained per IRB-approved protocol, but could have been programmatically purged from production databases upon participant request. Prior to participant recruitment, all technical safeguards (encryption, deidentification, and storage) were reviewed by the IRB as a part of a formal risk assessment. For ethical alignment with pediatric participants, access was granted only after parents and participants provided verbal and written consent. Per regulatory alignment for US minors, while Zotcare stores deidentified data (falling under “Safe Harbor” exclusions), it maintains technical safeguards, such as audit logs and AES-256 encryption, which are consistent with Health Insurance Portability and Accountability Act (HIPAA) Security Rule standards. Zotcare also supports all “Right to Know” and “Right to Delete” requests. Deletion could be executed via study participant number, ensuring the platform never required the participant’s identity to fulfill a request.

**Figure 2.**
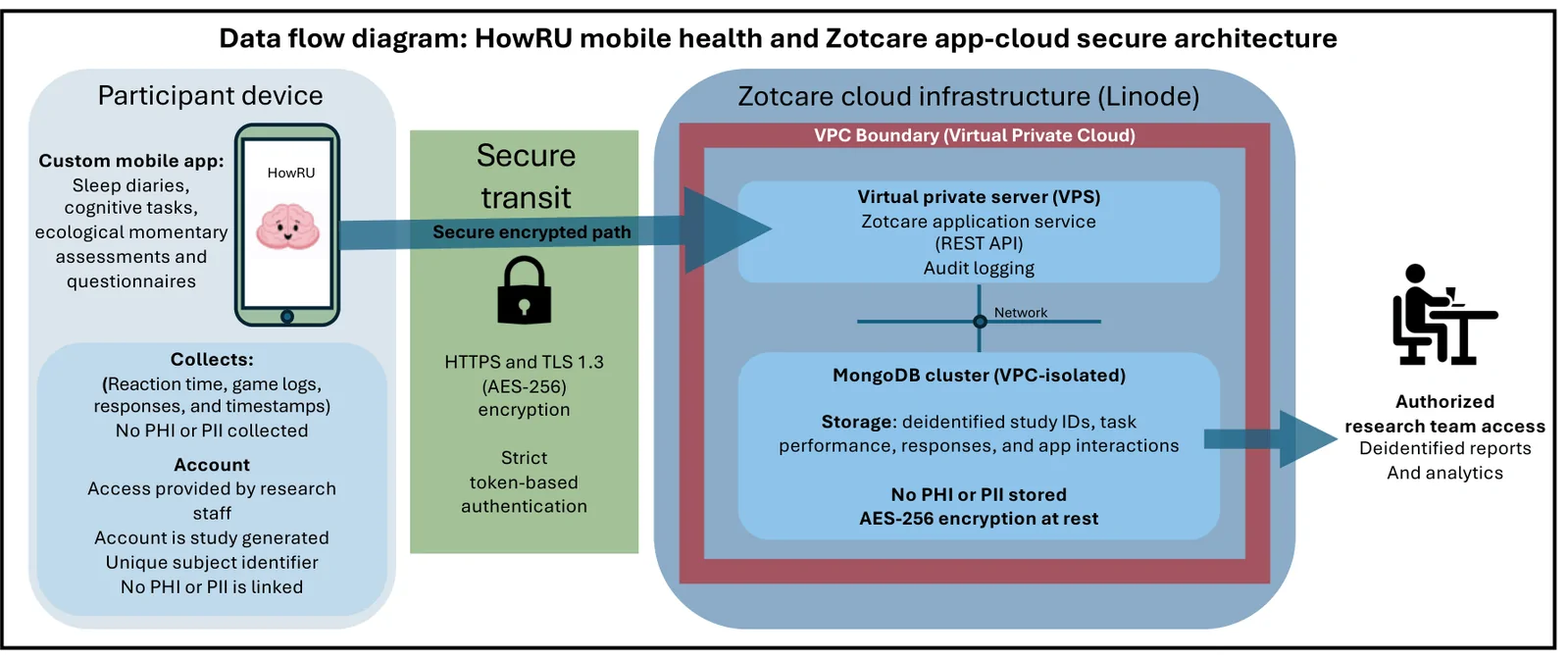
Schematic of mobile health platform HowRU and ZotCare platform data flow. AES: Advanced Encryption Standard; HTTPS: Hypertext Transfer Protocol Secure; PHI: protected health information; PII: personally identifiable information; REST API: Representational State Transfer API; TLS: Transport Layer Security; VPC: virtual private cloud; VPS: virtual private server.

### Baseline Testing

After participants and parents completed the consent and assent, participants were administered the Wechsler Abbreviated Scale of Intelligence (WASI) [51] vocabulary subtest and digit span for baseline functioning, the Questionnaire of Unpredictability in Childhood (QUIC) [52], and the depression and anxiety subsections of the Kiddie Schedule for Affective Disorders and Schizophrenia for School Aged Children – Present and Lifetime Version (K-SADS-PL) [53].

Baseline questionnaires were provided to parents and youth via REDCap links emailed after the consent session. Parents completed the following: a general demographics questionnaire; Pediatric Sleep Questionnaire (PSQ and SRBD), which measured sleep-related breathing disorder (SRBD) symptoms [54]; modified video game experience (VGE), which measured parent-reported perceptions of child VGE [55]; the Alabama Parenting Questionnaire (APQ)-Parent Report, which measured parent-reported use of positive and negative parenting behaviors [56]; the Parenting Self Agency Measure (PSAM), which measured parent beliefs related to their parenting abilities [57]; the COVID-19 Family Stressors Scale (FSS), which measured family stressors as a result of the COVID-19 pandemic [58]; and the COVID-19 Fear of Illness and Virus Evaluation (FIVE), which measured COVID-19–related fears in parents and youth [59].

Youth completed the following: APQ-Child Report for ages 6 to 13 years, which measured youths’ “perception of parents” use of positive and negative parenting behaviors [56]; Inventory of Parent and Peer Attachments-Revised (IPPA-R) for ages 9 to 15 years, which measured the quality of youth relationships with parents and peers [60]; Child and Adolescent Social Support Scale (CASSS), which measured youth and adolescent perceived social support from people in their life [61]; the Internet Addiction Test-Short Form (IAT-SF), which measured youth self-reported social media use and habits [62]; Difficulties in Emotional Regulation (DERS-SF), which measured emotional regulation in youth [63]; Morningness-Eveningness Questionnaire (MEQ), which measured morning and evening preferences in youth [64]; and Pittsburgh Sleep Quality Index (PSQI), which measured sleep behavior, quality, and disturbances in youth over the past month [65].

### Measurement Burst: Questionnaires

During each measurement burst (0, 4, 8, and 12 months), youth completed the following questionnaires once within the 7-day burst: (1) a paper-based Children’s Depression Inventory (CDI), which measured depressive symptoms in youth ages 7 to 17 years [66] and was mailed with the equipment at each burst and completed at home, and (2) using the HowRU app, youth completed the Center for Epidemiological Studies Depression Scale for Children (CES-DC), which measured levels of depressive symptoms in youth and adolescents ages 6 to 17 years [67]; the Adolescent Sleep-Awake Scale (ASWS), which assessed sleep quality [68]; the General Anxiety Disorder-7 (GAD-7), which measured symptoms of anxiety [69]; the Inclusion of Self and Others (ISO), which measured how close youths felt to their parents [70]; and the IAT-SF, which measured internet use [62]. Youth were requested to complete the Perceived Stress Scale (PSS), which measured youth’s perceived stress on the last day of the burst and reported their perceived stress during the course of that week [71]. Together, these measures provided the information necessary to assess youth’s report of their sleep behaviors or disturbances, depressive symptoms, and perceptions of their parents’ parenting behaviors, which could be linked to their control of sleep activities. Youth also reported pubertal onset and progression at each burst with the Pubertal Development Scale (PDS) [72]. The PDS is a validated measure for sex-specific physical development changes (eg, hair growth, breast development, and voice changes) associated with puberty. Based on self-report, youth were categorized into 1 of the 5 specific pubertal stages, ranging from prepubertal to postpubertal. Menarche and menstrual cycle frequency were also reported by female participants. Puberty status will be used as a time-varying covariate in analyses.

Parents completed the following: a paper-based Child Behavior Checklist (CBCL), which measured parents’ identification of emotional, behavioral, and/or social competence or adaptation problems in youth aged 6 to 18 years [73], and a paper-based Children’s Depression Inventory Parent (CDI-Parent), which measured parent perception of depressive symptoms in youth aged 7 to 17 years [66]. The paper-based CBCL and CDI were included in the mailed equipment box at each burst, and returned with the equipment at the end of each burst. Parents also completed the following questionnaires on HowRU (or via RedCap [Vanderbilt University] if preferred): the Children’s Sleep Habit Questionnaire (CSHQ), which measured sleep patterns and behaviors for 7- to 13-year-olds [74]; the Inclusion of Self and Child (ISC); and an adapted version of the VGE, which measured parent-reported perceptions of child VGE [55]. Together, these measures provided the information to assess parents’ identification of youths’ sleep behaviors and disturbances, internal and external symptoms, and their own parenting behaviors, which could be linked to their youth’s control of sleep activities.

### Measurement Burst: Sleep Diaries

All sleep diaries were administered via HowRU. Each morning, participants completed a sleep diary reporting bedtime, wake time, sleep-onset latency, arousals, and sleep quality. Participants also reported if they remembered dreams and, if so, the valence of the dream. At night, participants completed a sleep diary in which they reported if they napped (if so, for how long), the intentionality of the nap, dream occurrence, and dream valence. In the night diary, participants also reported the time spent on social media and associated mood.

### Measurement Burst: EMAs

All EMAs were administered via HowRU thrice daily: after waking up in the morning, between 3 and 5 PM in the afternoon, and prior to bedtime. EMA reminders were set at each measurement burst according to the participants’ schedule; for example, if an after-school activity ended at 4 PM, the notification window was moved from 4 to 6 PM to accommodate the change. These time points were chosen for feasibility and participants’ mobile technology access and were adequate for statistical modeling of affect variability within a day. App reminders were sent until completion and set with respect to the participant’s time zone (Pacific Standard Time, Mountain Time, Central Time, and Eastern Standard Time). All app reminders were flexibly set, that is, we aimed to send the morning reminder approximately 30 minutes after a participant’s typical wake-up time and 1 hour prior to typical bedtime. On the EMA, participants reported their current location, any individuals present at the time of the EMA completion, and their real-time emotions using the Positive and Negative Affect Scale ratings of excited, mad, sad, focused, guilty, happy, confident, and scared on a scale from 1 (not at all) to 100 (a lot) [75]. Participants also reported their highest level of stress in the last hour on the same 1-to-100 Likert scale and briefly responded using free text to explain what was on their mind at the time.

### Measurement Burst: Cognitive Tasks

#### Overview

We administered 5 cognitive tasks spanning cognitive domains of hippocampal-dependent, hippocampal-independent, and executive function. Four of the tasks were administered via HowRU**,** including the word-pair associates (WPA), motor sequence finger tapping (MST), rule switch (RST), and operation span (OSpan) task. The final task, the MMN, was tested separately on participants’ devices. For all the cognitive tasks, stimuli were counterbalanced across study days, measurement bursts, and participants. As practice effects were possible given the frequency of task administration, both the study day and measurement burst will be included in all future statistical analyses.

#### WPA

The WPA is a hippocampal-dependent memory task shown to be sleep-dependent in children and supported specifically by NREM [76]. The WPA consisted of learning and retaining 30 unique word pairs comprised of neutral, common words. Participants were trained on the word pairs in a single training block. Participants were tested on the word pairs twice, immediately after learning and once the following morning. At the time of testing, half the word pairs were re-presented in correct pairings while the other half were presented with alternative pairings. Presentation of the word pairs was randomized. For example, participants were trained on word pairs (1) cat-dog and (2) mouse-fish. Correct pairings could be “cat-dog” or “dog-cat,” but “cat-mouse” would be an inaccurate pairing. For this task, performance was measured as the difference in recognition accuracy between immediate and delayed testing. Unique word pairs were presented each night, such that no word could be seen twice throughout the entire study. Word pairs were counterbalanced across days and measurement bursts across participants.

#### MST

The MST is a hippocampal-independent task where neither youth nor depressed adults show sleep-dependent memory improvement [77]. The MST consists of learning and repeating a 5-digit sequence (eg, 4-2-3-2-1), on a panel of two-dimensional boxes, as efficiently and accurately as possible with the nondominant hand. Participants performed twelve 30-second trials of finger-tapping the sequence, with a 30-second break between training trials. Testing of the sequence occurred approximately 20 minutes after learning and again the following morning. Testing consisted of 4 trials in which the sequence was repeatedly typed for 30-second intervals, with 30-second rest intervals in between. For this task, performance was measured as the difference in accuracy and speed of pattern replication between immediate and delayed testing. For each night of the study, a unique sequence was presented to participants, so that they learned and were tested on 28 unique 5-digit sequences over the year. All sequences were counterbalanced across days and measurement bursts across participants.

#### RST

The RST is a hippocampal-independent task that evaluates cognitive processing, inhibition, and switching [78]. In the task, participants were trained to associate a specific food emoji with a congruent or incongruent box-click response. Performance within a session was assessed as the difference between congruent reaction time (ie, button presses) in the initial test when presented in isolation and reaction time during the mixed test, when the incongruent fruit emoji was intermixed in the presentation. For this task, accuracy and reaction time were compared between immediate and next-morning testing. Food emojis were randomized across testing sessions and measurement bursts, with none repeated for a participant within a year.

#### OSpan

##### The OSpan Task

The OSpan task assesses working memory [79]. Participants were presented with a series of letters in isolation and asked to retain them in their working memory in the order they were presented. Between each letter was a simple math equation and participants responded whether the equation was correct or incorrect. These trials of letter learning included letter lengths between 4 and 8, and thus an equivalent number of mathematical equations intermixed. For this task, performance was measured in accuracy of the trial letter sequence and compared between nighttime and next-morning performance. For all participants, letters were randomized within each trial.

##### The MMN Task

The MMN task is a spatial memory and navigation task that is assumed to be supported by the hippocampus [80,81]. For detailed methodology, please read Simon et al (2022) [80]. Briefly, participants learned the locations of 12 objects within a 3-D open-field Minecraft environment. Participants learned the locations across 2 training trials in which they were allowed free exploration. They were then tested on object location and their ability to navigate to these locations at 3 time points: immediately, the following morning, and the last morning (day 7) of the measurement burst. At test, participants were randomly teleported to an outer edge of the environment and provided an object to place. For this task, performance was measured as the difference in memory accuracy between immediate and delayed testing. Throughout the year, participants learned and were tested in a single environment at each measurement burst, with the environment order counterbalanced across participants and measurement bursts.

### Measurement Burst: Wearable Devices to Measure of Sleep and Wake Patterns and Neurophysiology

#### Overview

Participants wore a Garmin VivoSmart 5 continuously (day and night) for 7-days during each measurement burst [82,83]. At the start of each burst, research staff trained participants and parents to wear the watch on the nondominant wrist and to synchronize the data concurrently with the Garmin and LabFront apps using the study-provided tablets with both apps preinstalled. Youth were asked to wear the watch all day and night, removing it only with parent supervision during dinner time to charge it, and then replacing it to ensure complete data collection across the week and to minimize equipment loss. All data were collected in deidentified form, with each participant assigned a unique subject identifier. Raw data (eg, step counts and individual heartbeats) were extracted from the watch and accessed via LabFront, a software platform that provides raw time-stamped data. LabFront complies with International Council for Harmonisation of Technical Requirements for Pharmaceuticals for Human Use – Good Clinical Practice (ICH-GCP), HIPAA, Personal Information Protection and Electronic Documents Act (PIPEDA), General Data Protection Regulation (GDPR), ISO 27001, and ISO 9001 standards. Only participants and legal guardians who consented to the study were granted access to LabFront using a unique, deidentified subject identification number. No protected health information was collected or stored on the platform, and researchers maintained the link between identifiers and numbers. System administrators could access the infrastructure for algorithm deployment or maintenance but were unable to reidentify participants. All data could similarly be removed upon request and using the unique subject identification number. In addition to LabFront’s built-in algorithms, heart rate variability was derived from the raw interbeat interval data in accordance with the Task Force guidelines [84]. Garmin devices have been validated in pediatric samples of comparable age for assessing step count and physical activity [85] but have been found to overestimate sleep onset latency and underestimate sleep offset [86]. As such, Garmin data were primarily used to evaluate physical activity, while participants also wore sleep electroencephalography devices for more precise assessment of sleep neurophysiology and timing. LabFront’s proprietary algorithms, which yielded estimates of sleep (eg, duration, arousals) and physical activity, are comparable to research-grade Philips Respironics Actiwatch [82,83]. Wear time was calculated from the raw heart rate data, with nonwear time defined as consisting of at least 5 consecutive minutes during which time no heartbeats are detected.

Participants were also asked to wear an Interaxon MuseS EEG headband (second generation) to monitor sleeping brain rhythms. Muse headbands provided a noninvasive validated measurement of sleeping brain rhythms, allowing us to quantify the total amount of sleep stages and arousals across the night, measure the spectral power of the sleeping brain rhythms, and provide raw data to evaluate sleep rhythms, including slow wave sleep, a valid biomarker for depression [87-89]. These headbands were worn and able to be placed by parents and youth at home. Parents and youth were trained to assist with the placement of the wearables, with the Muse worn only at night and charged during the day. MuseS is a consumer product, not medical grade equipment. Paralleling Zotcare and LabFront, only participants and legal guardians who consented to the study were provided access to the Interaxon Muse platform and temporarily provided equipment. The data were collected using the deidentified unique subject identifier, and no protected health information was collected or stored on the platform. The link between participant and number was maintained by researchers, and Muse could not access personally identifiable information. System administrators could access the infrastructure for algorithm deployment or maintenance but could not reidentify participants. All data similarly could be removed by request, and through the unique subject identification number.

The Interaxon Muse headbands consist of 2 frontal channels (AF7 and AF8) and 2 temporal channels (TP9 and TP10). Raw sleep EEG was collected in 30-second epochs and proprietary algorithms were applied to the data to provide a sleep staging score. This scoring aligns with American Academy of Sleep Medicine scoring and was validated against gold-standard polysomnography in adults [90] and more recently used in 10-consecutive nights in college students [91]. In future analyses, we plan to assess the following microarchitecture features: total sleep time, total time spent in each sleep stage, percent and relative time spent in each stage, sleep onset latency, sleep stage latency, and sleep fragmentation including the total number of arousals and time spent awake after sleep onset. We also plan to characterize spectral power across all frequency bands within each sleep stage, including slow wave sleep (1‐4Hz), theta (4‐8Hz), alpha (8‐12Hz), sigma (12‐16Hz).

#### Data Quality

All initial task sessions were administered remotely via teleconferencing (eg, Zoom Communications Inc, Zoom). Research staff conducted an initial training session to instruct participants on the use study devices, the mHealth app, and cognitive tasks. Training included practice trials for each task with trial-specific feedback to ensure comprehension. Participants were verbally queried to confirm task understanding and retrained if necessary. Practice data were reviewed to verify that the task requirements were met before participants proceeded to the experimental session. Following the initial session, participants completed tasks independently, with research staff available on call for assistance. Task completion was monitored daily, and parents and youth were contacted if task data were missing or incomplete. Attention was assessed through explicit verbal checks during Zoom sessions and by monitoring reaction time during cognitive tasks and app interactions to identify lapses in participation.

To further enhance data quality, participants used study-provided devices prepared and maintained by the research staff, which were sanitized between participant use. The devices were configured to allow only study-related apps, and all other apps and internet-related functions were restricted. Participants were trained on device use during the initial session, including a demonstration by research staff, and were then required to demonstrate independent navigation of the apps. Participants were eligible for a single retest under predefined conditions of documented technical disruption (eg, connectivity loss). Retesting used novel stimulus sets, distinct from those previously administered. In cases of partial data loss, only valid task segments were retained.

### Risk Monitoring

As this was a remote research study involving youth who could experience depressive symptoms, the protocol included specific procedures to ensure participant safety. First, all participants completed oral and written informed consent and assent. During the consent process and prior to signing, research staff clearly explained the limitations of confidentiality, noting that confidentiality was maintained except unless information was disclosed by the youth or parent indicating risk of harm to self or others (including suicidality, homicidality, child abuse, or neglect). Participants and parents were also informed that parents would be notified if assessments indicated moderate or severe levels of depression. If safety concerns arose through self-disclosure, parent disclosure, or questionnaire response, a licensed psychologist conducted a risk assessment (including evaluation of suicidal or homicidal ideation, plan, or intent), engaged in safety planning, and provided appropriate behavioral health referrals. Had an imminent safety risk been present, emergency services would have been contacted for further assessment and support. If child abuse or neglect was disclosed, a report would have been filed with California Child Protection Services, consistent with mandated reporting requirements. Documentation of all risk assessments was maintained in confidential, password-protected files only accessible by the licensed psychologist. Daily mood and sleep diaries did not assess self-harm or suicidal ideation. These topics were instead evaluated during the initial administration of the K-SADS-PL and subsequently, during assessment waves in the Child Depression Inventory. As the latter questionnaire was mailed to and returned by participants, it was reviewed immediately upon receiving the equipment box. Finally, if participants reported symptoms consistent with moderate to severe depressive symptoms on the questionnaires, parents were contacted by phone and email to discuss the results and were provided with mental health resources.

### Statistical Analysis

#### Sample Size

Power analyses were conducted in Heinrich Heine University Düsseldorf G*Power to provide a conservative benchmark using repeated measures ANOVA sensitivity analyses [92]. Assuming 4 measurement bursts of data and ignoring the intensive, within-burst repeated observations, a sample of n=27 provided 80% power to detect moderate within-person effects on memory of approximately Cohen *f*=0.3. These estimates were conservative relative to the proposed analytic plan. Future primary analyses will use multilevel modeling, latent growth curve modeling, and structural equation modeling, which model within-person variability across repeated observations (7 to 21 measures within each burst) and reduce measurement error through latent-variable estimation. We did not assume a uniformly greater power, as the effective power gain depends on the number of participants and the proportion of within-person variance captured. In our models, we will use full-information maximum likelihood (FIML), which allows for the inclusion of incomplete data under the assumption that the data are missing at random. We anticipated 70%‐80% adherence in the cognitive task and sleep diary completion within each burst, based on prior longitudinal studies. As such, we anticipated enough observations collected per participant to detect within-person effects. Given these considerations and our conservative approach, the targeted sample size of 27 participants, evenly distributed between ages 9 to 13 years, was expected to provide adequate power. To account for attrition, equipment malfunction, or missing data, we recruited additional participants, for a total recruitment of 56 youth, which ensured that we had sufficient data for the planned multilevel and latent variable analyses.

#### Analysis Approach

In our future planned analyses, we will first conduct descriptive analyses to characterize the distributions, central tendencies, variability, summary statistics, and within- and between-person correlations. We will then determine data quality using visual inspection and diagnostic statistics to identify possible outliers or anomalous observations. As necessary, outliers will be examined and addressed using principled approaches, specifically sensitivity analyses or robust estimation.

Primary analyses will use multilevel modeling (MLM) approaches with random intercepts at the participant level and, when supported by model fit, will include random slopes as key predictors. Time-varying covariates will include age, day of the week, day of the study week, school versus nonschool day, and measurement burst, and will be modeled at the within-person level. Nontime-varying covariates, including sex, pubertal status, and socioeconomic status, will be included at the between-person level. We will also use FIML, which allows the inclusion of incomplete data assumed missing at random. We will also model pubertal effects continuously and nonlinearly by incorporating pubertal status and age-by-puberty interaction terms into the longitudinal models. These will allow us to test whether sleep-depression-cognition associations change across developmental stage or age. Primary outcome analyses are described below for the main study aims. Secondary and exploratory study analyses are identified and will be interpreted with caution as our study may be underpowered to detect smaller effects. We will use false discovery rate procedures for secondary and exploratory outcomes.

### Primary Outcomes

The primary outcomes of this study are to (1) characterize sleep and depressive symptom trajectories across the year and (2) determine the relationship between sleep and depressive symptoms on sleep-dependent cognitive performance across the year.

### Data Analysis Plan for Primary Outcomes

To characterize sleep and depression trajectories, we will use multilevel models to characterize the changes in sleep and depressive symptoms across measurement bursts. The primary contrasts of interest are the between-person differences in sleep and depressive symptom change over time. Our planned contrasts are to (1) evaluate the mean slope of sleep duration and depressive symptoms across measurement bursts, (2) determine the presence of nonlinear change (acceleration or deceleration) with age and pubertal status, including the use of quadratic terms to determine the rate of change, and (3) to analyze the covariance between the intercepts and slopes of sleep duration, sleep variability, and depressive symptoms. Exploratory analyses will examine correlations between the intercepts and slopes over time, allowing evaluation of how correlated the longitudinal trajectories are.

To evaluate the longitudinal associations between sleep, depressive symptoms, and cognition, we will use multilevel models. The primary contrasts of interest are the within-person differences from each individual’s personal mean in sleep and cognitive performance. Planned contrasts are to test whether sleep duration predicts next-day sleep-dependent cognitive performance and the association with youth’s depressive symptom severity. We frame our hypotheses sequentially. First, we hypothesize that, relative to a participant’s average sleep metrics, a night of short sleep duration will predict worse next-day cognitive performance compared to that participant’s typical performance. If this effect is established, we will then test whether these effects are stronger among youth with higher depressive symptoms. Our main model also includes a mediation analysis component to test if insufficient sleep duration predicts worse sleep-dependent cognitive performance indirectly via elevated depressive symptoms over time. In parallel to the main model, we will consider 2 alternative specifications if the main model does not fit the data adequately: alternative model 1, in which depressive symptoms predict sleep-dependent cognitive performance deficits indirectly via insufficient sleep; and alternative model 2, which examines only the direct associations between insufficient sleep, depressive symptoms, and sleep-dependent cognitive performance. These competing models will be evaluated and compared using fit statistics. Distinguishing the most distal (early) predictor is key for informing future intervention efforts.

### Secondary Outcomes

Secondary outcomes include (1) the impact of sleep and depressive symptoms on the additional 2 cognitive tasks, the rule switch and motor sequence learning task, and (2) the parent and youth report of parenting factors on sleep patterns. Additional secondary outcomes will be explored, including changes in daily mood, heart rate variability, physical activity, and psychosocial questionnaires (eg, social media use, parenting behaviors, peer support, and parent-reported child behavioral and emotional patterns). Secondary and exploratory outcomes will be interpreted with caution as smaller effects may be underpowered.

## Results

### Timeline

Funding was obtained from the Eunice Kennedy Shriver National Institute of Child Health and Human Development (Grant: K08 HD107161) with a budget period of August 1, 2022, to July 31, 2027. Participant enrollment occurred from May 2023 to October 2024. Data collection began in July 2023 and was completed in October 2025.

### Participants, Recruitment, and Retention Results

Figure 3 displays participant adherence and retention rates across the study year from initial study screener contact through the final assessment wave. Fifty-six participants were enrolled in the study (female n=27). The average age was 11.01 (SD 1.39) years. From consent through the end of the last measurement burst, participant completion was 75% (42/56). Among those who successfully completed the first measurement burst, 86% (42/49) completed all 4 assessment weeks. Attrition was the highest between the first and second measurement bursts at 10.2% (5/49) and decreased between the second and third measurement bursts at 4.5% (2/44); attrition between the final third and fourth measurement bursts was 0% (42/42). These findings indicated strong participant retention over the year-long study, with greater confidence in retention among participants if they completed the first measurement burst.

**Figure 3.**
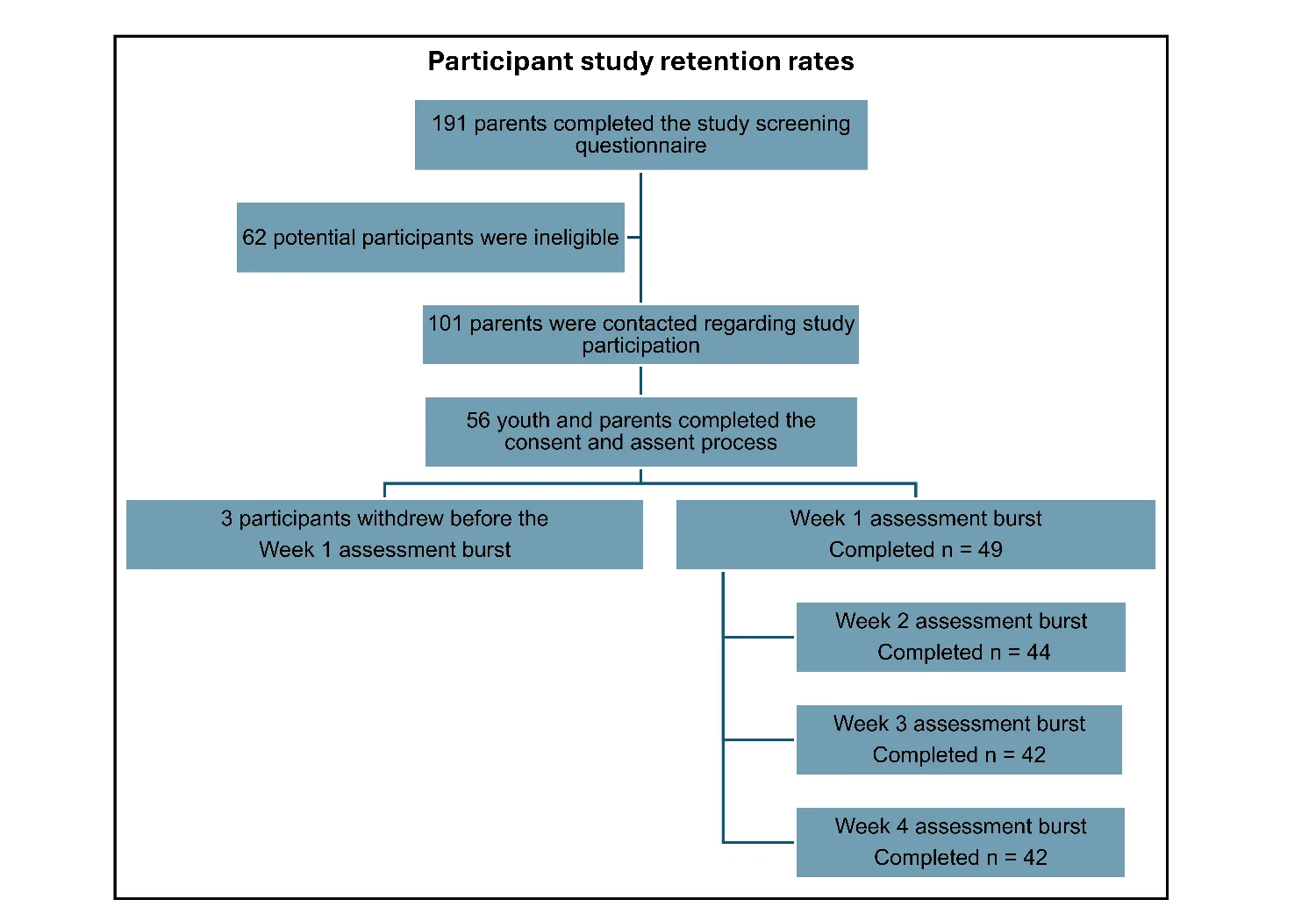
Participant adherence and attrition across the study. Following initial screening, 101 parents and youth were contacted regarding study participation. Of these, 56 youth and parents responded and completed the consent and assent process. Three participants withdrew after consent but before initiating the study. Fifty-three participants initiated the Week 1 assessment burst, of whom 49 completed Week 1. Following Week 1, five participants withdrew, and 44 completed the Week 2 assessment burst. Two additional participants withdrew prior to Week 3, resulting in 42 participants completing the Week 3 assessment burst. All 42 participants who completed Week 3 also completed the final Week 4 assessment burst.

### Protocol Adherence

Participants demonstrated high rates of adherence to cognitive tasks, sleep diaries, and EMAs. We have included results in Table 1 across the measurement bursts. Adherence was lowest for the EMA assessments, likely brought down by the afternoon EMA. In the MMN task, all missing data were primarily due to the lack of access to a computer that could host Minecraft (Mojang Studios) or due to a technical error that prevented Minecraft from working.

**Table 1.** Adherence rates for all data collection measures completed during a measurement burst.

Data collection measures	Measurement burst 1 (%), mean (SD)	Measurement burst 2 (%), mean (SD)	Measurement burst 3 (%), mean (SD)	Measurement burst 4 (%), mean (SD)
WPA^a^ task	91.03 (17.3)	94.9 (6.9)	91.07 (14.2)	91.07 (15.6)
OSpan^b^ task	89.9 (19.05)	89.8 (13)	87.6 (20.6)	86.9 (21.7)
RST^c^	90.7 (18.1)	94.4 (8.6)	89.8 (15.2)	91.6 (16.1)
MST^d^	91 (18.9)	94.5 (8.5)	91.6 (13.5)	91.1 (15.4)
MMN^e^ task	96	93	92.8	90.5
Sleep diaries	87.3 (22)	91.8 (13.6)	89.1 (15.6)	91.9 (14)
EMA^f^ assessments	80.4 (25.8)	77 (22.8)	71.7 (28.5)	80.4 (26.7)

a WPA: word-pair associates.

b OSpan: operation span.

c RST: rule switch task.

d MST: motor sequence finger tapping task.

e MMN: Minecraft memory and navigation.

f EMA: ecological momentary assessments.

## Discussion

### Principal Findings

The neural mechanisms linking depression-related cognitive impairments and sleep remain poorly understood. Given sleep’s dual role in supporting both mood regulation and cognition, sleep disturbances may represent a critical risk factor for long-term cognitive outcomes in youth with depression. However, precision medicine approaches to early-onset depression are still in their infancy, and few studies track candidate biomarkers longitudinally in real-world environments. To optimize early intervention time windows and targets, there is an urgent need to track real-world biomarkers that are dually linked to early-onset depression and cognitive impairments. Remote and accessible mHealth testing platforms are the future for longitudinal integrated investigations of clinical and cognitive health in adult as well as pediatric populations [93-95]. Technology access is reported in 67% to 78% of 9- to 11-year-olds [96]; thus, using a technological platform to investigate mechanistic questions of cognitive development has a strong potential for success.

Longitudinal, measurement burst designs are unusual in sleep and cognition research, where most investigators rely on short-term experiments of 1 day or a few weeks [47]. In contrast, the current study’s longitudinal design allows for the disentanglement of long-term trajectories from the relative contributions of sleep and depressive symptoms across different time scales throughout development. mHealth assessment increases access and participation feasibility and simplifies intensive repeated-testing procedures in youths [93-95]. Furthermore, wearables that track sleep patterns, physiology, and physical activity in naturalistic settings (eg, home and school) offer a robust platform for investigating the effects of interventions for depression in large-scale longitudinal studies [48,49]. Findings from this study will support the early detection of depression risk through sleep-related biosignals captured by accessible, cost-effective consumer wearable devices. The results may also identify critical time windows during which sleep-based interventions could help prevent the onset of mental health symptoms. Clinically, integrating consumer wearable devices offers a practical and scalable approach to monitoring physiological biomarkers associated with mental health, enabling practitioners or schools to implement effective, first-line screening and prevention strategies. Lastly, tracking cognitive performance across multiple timescales and its associations with sleep and mental health may provide novel insights into how cognitive skills can be more effectively targeted in therapy to alleviate depressive symptoms.

### Limitations and Future Directions

Study limitations were primarily due to the nature of our remote study design. First, participants completed the study remotely from their homes, meaning that research staff could not control the environment for noise or interruptions to the same degree as in-lab studies. To reduce this potential problem, we provided instructions to parents and youth to plan accordingly and ensure they were alone and focused, without distractions from phones, family, or friends, during participation. Further, we temporarily provided a study tablet to each participant to reduce measurement error caused by different devices or screen sizes. While these steps were taken to mitigate measurement error and vulnerability to noise, real-world data collection remains vulnerable to at-home conditions. A second limitation was that participant adherence to daily cognitive tasks, sleep diaries, and EMAs was lower than in lab participation. To combat data loss, participants’ app-based engagement was monitored daily by research staff. To reduce the loss of app-based data, personalized notifications for youth were timed to daily tasks and personal schedules. Reminders repeated every 30 minutes for 2 hours or until task completion. Research staff also monitored data collection daily, and if tasks were not completed, research staff contacted parents and participants via email with reminders to complete the daily tasks in the required time windows. Overall, our adherence to daily tasks was very high but was lowest for the EMA. We believe this is due to the afternoon EMA completion. Future studies can better target windows that participants are more likely to be near devices (ie, after school) or possibly enhance the incentive to complete the afternoon EMA. A third limitation in our study was the loss of wearable data due to equipment failure, incorrect device use, or no device wear. In this study, participants were asked to wear the Garmin VivoSmart 5 watch all day and night, and the MuseS headband each night, for the duration of the burst. Participants, with assistance from parents, were requested to sync the data daily. To increase the likelihood of strong data collection, at the start of each measurement burst, participants and parents met with research staff via Zoom and learned how to place and wear the devices, sync the data, and practiced wearing the devices in front of the staff to confirm the fit was correct. Further, during the week, research staff monitored daily uploads of wearable data, including the total daily and nightly wear time of the devices and any data loss due to poor wear. Emails were sent to parents to remind the participants to wear the devices, and additional Zoom-based meetings were conducted as needed to assess fit and improve adherence. This increased our data collection adherence and the accuracy of wearable biosignal data. Lastly, we administered the MEQ and the IAT-SF, which were both validated in young adults and have limited validity in children, limiting our ability to accurately determine participant chronotype and internet use. Future directions in longitudinal remote research can expand on the sample population to include youth with common comorbid mental health and sleep disorders. As we excluded youth participants with other neurobehavioral and sleep disorders, the generalizability and external validity of our findings will be somewhat limited to those with depressive symptoms. However, this trade-off will allow us to examine sleep, mood, and cognitive performance trajectories within a more homogenous sample, reducing confounds from co-occurring symptoms. Future studies could broaden the age range to include older adolescents and incorporate longer longitudinal follow-up periods.

### Conclusion

Depressive symptoms that emerge in early adolescence can lead to long-term psychological, physical, and cognitive consequences. Given that poor sleep is predictive of depression onset and symptom severity, and a primary symptom of depression itself, understanding how sleep behavior and neurophysiology predict early depressive symptoms may be key in understanding adolescent depression. Further, depressive symptoms and insufficient sleep can lead to cognitive performance deficits. Anticipated contributions from this protocol include a roadmap for longitudinally tracking sleep, depressive symptoms, and cognitive trajectories, with the aim of providing critical insights and identifying essential time periods for proactive intervention. Additionally, results from this study will also help determine which sleep behavior or neurophysiological metric offers the most promising target for altering mental health and cognitive trajectories. By tracking changes in sleep alongside mental health over time, we anticipate revealing how their dynamic interplay influences cognitive development. Our protocol is a robust and reproducible framework for tracking young adolescents over time using mobile health platforms and wearable technology, sleep monitoring, mental health assessments, and cognitive processes. This study will help fill a critical gap in understanding adolescent day-to-day variability in the real world.

## Supplementary material

10.2196/86345 Checklist 1 STROBE (Strengthening the Reporting of Observational Studies in Epidemiology) Checklist

## Abbreviations

AES: Advanced Encryption Standard
APQ: Alabama Parenting Questionnaire
ASWS: Adolescent Sleep-Awake Scale
CASSS: Child and Adolescent Social Support Scale
CBCL: Child Behavior Checklist
CDI: Children’s Depression Inventory
CDI-Parent: Children’s Depression Inventory Parent
CES-DC: Center for Epidemiological Studies Depression Scale for Children
CSHQ: Children’s Sleep Habit Questionnaire
DERS-SF: Difficulties in Emotional Regulation
EEG: electroencephalogram
EMA: ecological momentary assessment
FIML: full-information maximum likelihood
FIVE: fear of illness and virus evaluation
FSS: Family Stressors Scale
GAD-7: General Anxiety Disorder-7
GDPR: General Data Protection Regulation
HIPAA: Health Insurance Portability and Accountability Act
IAT-SF: Internet Addiction Test-Short Form
ICH-GCP: International Council for Harmonisation of Technical Requirements for Pharmaceuticals for Human Use – Good Clinical Practice
IPPA-R: Inventory of Parent and Peer Attachments-Revised
IRB: Institutional Review Board
ISC: inclusion of self and child
ISO: inclusion of self and others
K-SADS-PL: Kiddie Schedule for Affective Disorders and Schizophrenia for School Aged Children – Present and Lifetime Version
MEQ: Morningness-Eveningness Questionnaire
mHealth: mobile health
MLM: multilevel modeling
MMN: Minecraft Memory and Navigation
MST: Motor sequence finger-tapping task
NREM: nonrapid eye movement
OSpan: operation span
PDS: Pubertal Development Scale
PIPEDA: Personal Information Protection and Electronic Documents Act
PSAM: Parenting Self Agency Measure
PSQ: Pediatric Sleep Questionnaire
PSQI: Pittsburgh Sleep Quality Index
PSS: Perceived Stress Scale
QUIC: Questionnaire of Unpredictability in Childhood
RST: Rule Switch task
SRBD: sleep-related breathing disorder
STROBE: Strengthening the Reporting of Observational Studies in Epidemiology
TLS: Transport Layer Security
VGE: video game experience
VPC: virtual private cloud
WASI: Wechsler Abbreviated Scale of Intelligence
WPA: Word-pair associates
Zero-PHI: zero-protected health information policy
